# A novel personal identification system using doorknob lead electrocardiograms for unconscious authentication in unlocking doors

**DOI:** 10.3389/fdgth.2025.1585431

**Published:** 2025-06-20

**Authors:** Keisuke Kawamura, Masaki Kyoso

**Affiliations:** Graduate School of Integrative Science and Engineering, Biomedical Instrumentation Engineering Lab, Tokyo City University, Tokyo, Japan

**Keywords:** biometric authentication, ECG, synchronized averaging, machine learning technique, artificial neural network, support vector machine, data augmentation

## Abstract

**Introduction:**

In highly information-oriented society, personal authentication technology is essential. Biometric authentication is becoming popular as a method of personal authentication from the viewpoint of usability. In this research, in order to realize unconscious personal authentication during daily activities, we proposed a novel biometric authentication system using a doorknob-type electrocardiogram (ECG) measuring device. In our previous study, it was shown that ECG obtained with a contact-type electrode on doorknob and a capacitive-type electrode on the floor could be used for personal identification. However, identification performance is easily affected by noise from body movements and other factors, due to loose contact between electrodes and the body.

**Method:**

In this paper, we proposed to add two preprocessing techniques to the system. Synchronized averaging process was applied to the measured ECG waveforms. Then, data augmentation was applied to the machine learning training data.

**Results:**

It was found that synchronized averaging with 5 consecutive wave segment improved accuracy by 10%. It was also found that training data augmentation improved the performance even under limited amount of ECG data.

**Discussion:**

The results demonstrate that remarkable performance improvement can be achieved even with short term door-knob ECG by using synchronized averaging and data augmentation.

## 1 Introduction

In a highly information-oriented society, personal authentication technology is essential. Biometric authentication is becoming popular as a method of personal authentication from the viewpoint of usability. Biometric authentication is currently the mainstream method of personal identification (1). It uses the physical and behavioral characteristics of the user to verify his or her identity. Therefore, it is less burdensome for the user because there is no need to carry or memorize the information, and it is superior to conventional methods. However, there is a risk of information leakage and the inability to change the information once it has been leaked. Among biometric methods, personal authentication using biometric signals is considered superior in security because it is difficult to duplicate from the outside. Electrocardiogram (ECG) can be used for a novel secured biometric feature (2–8). In these previous studies, some feature parameters extracted from ECG were analyzed by multivariate statistical analyses or traditional classification techniques. The ECG is superior among biological signals because its signal is relatively large and can be acquired more easily than other biological signals.

Furthermore, heart disease is the second leading cause of death in Japan (9). Therefore, daily healthcare at home is considered necessary. Capacitive electrodes are effective for the unselfconscious measurement of electrocardiograms (10, 11). The introduction of such a system would create an environment in which ECGs can be measured unselfconsciously and the data can be automatically classified in households with two or more members.

We wondered if ECG could be used to unlock doors in a home environment where such an automatic ECG measurement and management system is installed. This would create an environment in which doors can be unlocked unconsciously. The system not only manages entry to rooms but also knows who is using the room. In ECG personal identification, we have been working on high-frequency components in ECG (HFECG) (12–14). We have shown that the extracted individual features give high identification performance. In these previous studies, we have used machine learning techniques such as artificial neural networks to deal with fluctuating features. We have also used doorknob-type electrodes which consist of a contact-type doorknob and capacitive-type floor electrodes (15). The signal quality from the electrodes is far inferior to the quality of contact-type electrodes. Therefore, basic machine learning identification had limited performance due to noise, which is inevitable in the practical environment of capacitive electrodes. In this paper, as a proposal for improvement, we introduced the synchronized averaging method used for noise reduction in the field of EEG analysis to signal preprocessing and added a device to expand the number of training data for machine learning to improve performance. The same discriminators were used for machine learning to compare the results with those of previous studies. Verification of the discrimination accuracy suggested that it is possible to discriminate with an accuracy of >95% even when noise is mixed in under practical conditions, and results that bring the system closer to practical use were obtained.

## 2 Method

### 2.1 ECG measurement

ECG data were measured from 10 healthy subjects consisting of seven males and three females with an average age of 21.2 years in a shielded room. The number of subjects was determined based on the assumption of home use. Five sets of measurements were performed for 20 s each. ECGs were recorded both with a doorknob ECG measuring device and with disposable electrodes for comparison. The total measurement time was approximately 10 min per person, and the subjects were in a resting state, so the measurement could be performed while the ECG remained steady.

**Figure 1:**
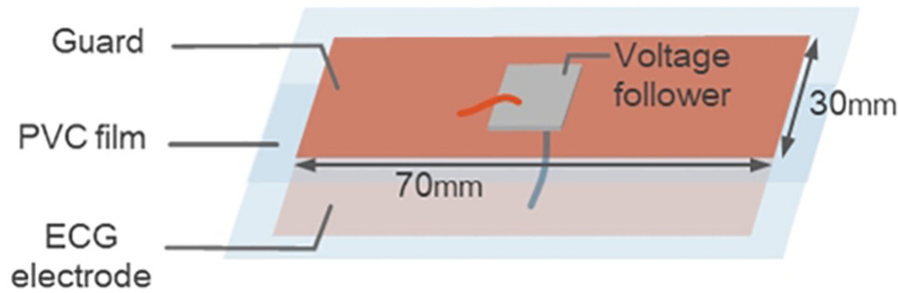
Floor electrode.

The doorknob-type electrocardiograph consists of a doorknob electrode and two floor electrodes as shown in Figure 1. The doorknob electrode is a contact electrode that consists of a gold-plated copper foil affixed to a doorknob. The floor electrodes are capacitive electrodes insulated with 0.07 mm polyvinyl chloride (PVC) film attached to both sides of the copper foil, and a voltage follower is attached to the back of the electrode to reduce the output impedance. A copper foil was used to ensure flexibility and conductivity to fit the shape of the sole, while PVC film was employed because of its dielectric constant, thickness, and flexibility to ensure sufficient capacitance with the living body. The floor electrodes were placed on a urethane mat to improve adhesion to the floor electrodes to ensure close contact between the electrode and the sole. Flexible electrodes allow for better adhesion to the plantar surface and stable measurement.

**Figure 2:**
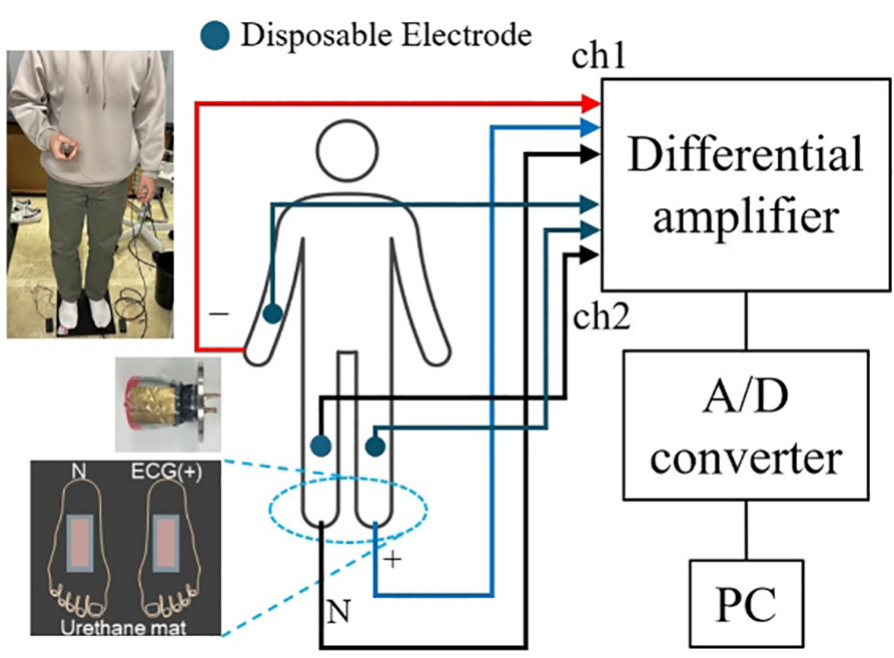
Measurement system configuration.

Doorknob ECG was recorded in a standing position, with the doorknob electrode connected to the negative input terminal, the floor electrode on the left foot side connected to the positive input terminal, and the floor electrode on the right foot side connected to the ground, as in limb lead II. As shown in Figure 2, the subject was instructed to hold the doorknob electrode with his right hand and to stand on the floor electrodes with his socks on so that he is in a posture simulating the motion of opening a door. Measurements with disposable electrodes were performed with limb lead II using the wrist and ankle simultaneously. Two signal grounds were separated from each other so that the ground for limb lead II connected directly to the body does not have any effect on the doorknob ECG. The measurement environment and conditions are shown in Figure 2 and Table 1. The measuring instrument used was the AB-610J manufactured by Nihon Kohden Corporation. The results of the measurement waveforms using the disposable electrodes are referred to as “Dispo,” and the results of the measurement waveforms using the doorknob-type electrocardiograph are referred to as “Door.”

**Table 1:** Measurement parameters.

Parameters	
Sampling frequency	1,000 Hz
High pass filter cutoff frequency	0.08 Hz
Low pass filter cutoff frequency	300 Hz
Hum filter cutoff frequency	50 Hz
Gain	60 dB
Resolution	16 bit
Input voltage range	±5 V

Additional measurements were performed on 7 subjects consisting of five males and two females selected from the 10 subjects mentioned earlier on other days under the same conditions. These data were used for reproducibility evaluation. The system was trained using the data of the first 10 people, and these data were then used as test data to evaluate the performance.

All the measurements were performed with careful ethical consideration and approval by the Medical Ethics Committee of Tokyo City University.

### 2.2 ECG preprocessing

#### 2.2.1 Outline

**Figure 3:**
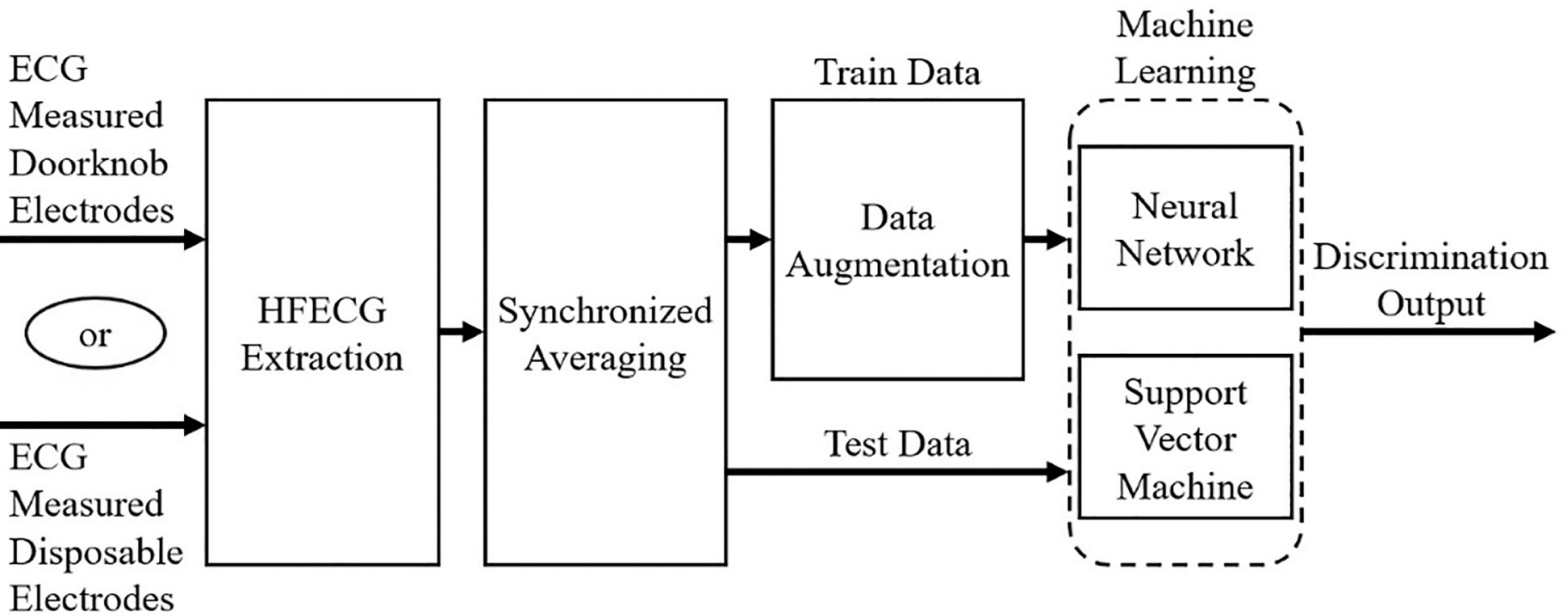
Overall ECG processing flow.

Figure 3 shows the overall processing flow. As shown in the figure, 200 samples of the measured ECG waveform were cut out based on the peak on the R wave. Then, synchronized averaging was performed on the extracted ECG waveforms, and appropriate extensions were applied to the obtained waveforms to create training data. For the training data, the waveforms averaged 1 or 2 of wave segments were used for data expansion. Using this waveform data, machine learning was performed to classify subjects.

#### 2.2.2 Beat-by-beat HFECG extraction

**Figure 4:**
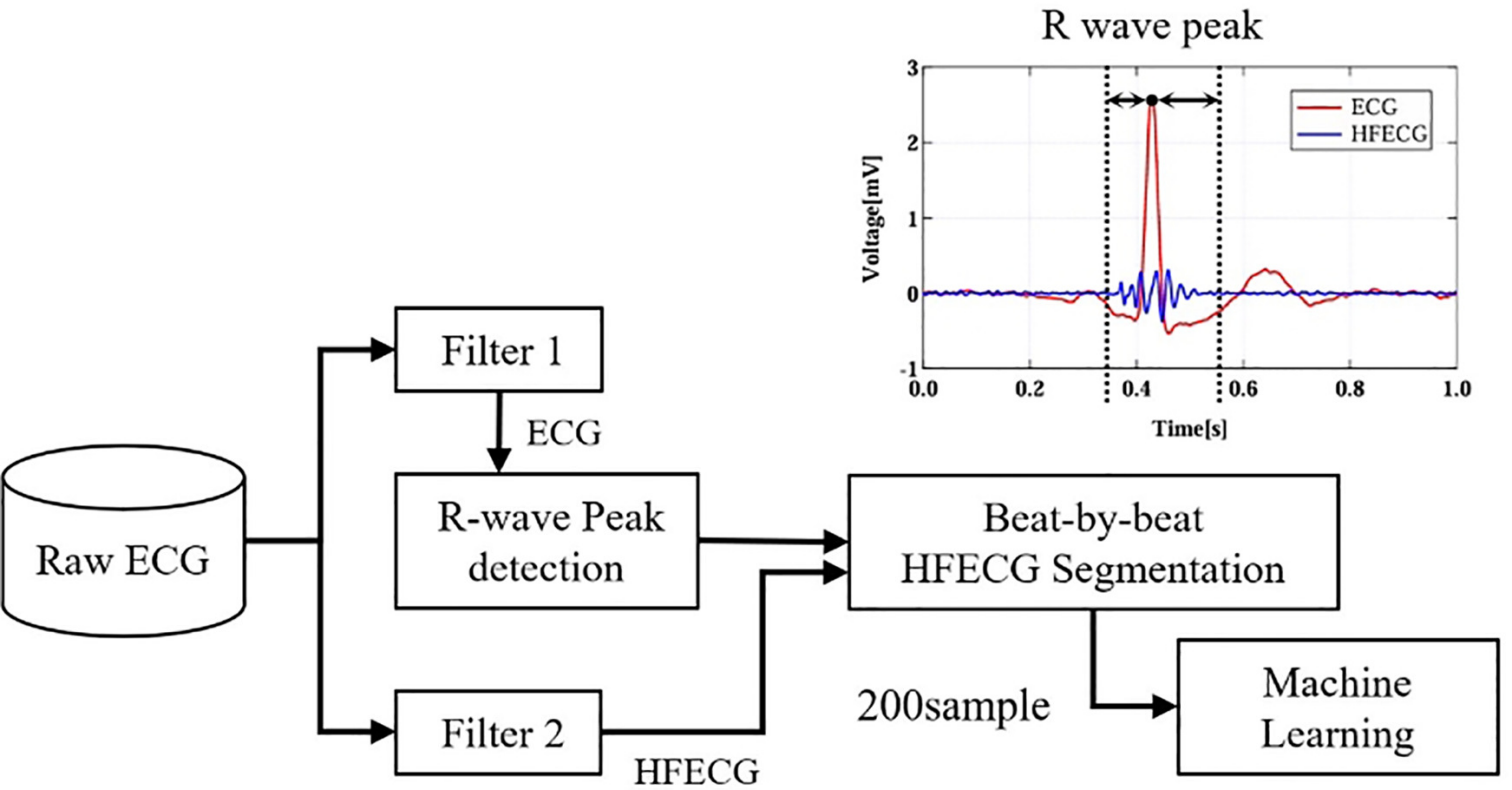
HFECG extraction flow.

The extraction flow of the high-frequency ECG is shown in Figure 4. Segmentation parameters were determined by preliminary experiments and previous studies (12–14) so that the high-frequency ECG corresponding to the QRS wave could be covered in any subject. Filter 1 and Filter 2 were used to obtain ECG and HFECG, respectively. A total of 200 samples were extracted from the HFECGs after Filter 2. The digital filter is an IIR filter with Butterworth characteristics. The wave segment consists of 75 samples before the R wave peak is detected by Filter 1 as the range into which the main waveform enters and 125 samples after it is detected (12). This resulted in a 0.2 s extracted around the QRS wave of a single heartbeat waveform. The filter conditions are shown in Table 2. Waveform samples were used as personal features input to the discriminator.

**Table 2:** Filter settings.

Filters		Cutoff frequency	Order
Filter 1	High pass filter	0.5 Hz	4
	Low pass filter	150 Hz	12
Filter 2	High pass filter	40 Hz	4
	Low pass filter	150 Hz	12

#### 2.2.3 Noise reduction by synchronized averaging

In usability-oriented ECG measurements such as doorknob electrodes, the contact between the skin surface and the electrode is uncertain. It results in unavoidable noise contamination, which has a negative impact on discrimination performance.

As a method to remove noise while preserving waveform characteristics, this study proposes the application of synchronized averaging, which is also used in evoked brain potential measurements (16). Synchronized averaging is a method used mainly when the target waveform in a signal is weak or when the signal is polluted by noise that has the same frequency component. Consecutive signals are cut out at the same timing and then added and averaged. This has the advantage of noise reduction and target waveform enhancement. This process improves the signal-to-noise ratio by a factor of $\sqrt{n}$ because it makes the signal enhanced by *n* times and uncorrelated signal enhanced by $\sqrt{n}$ times. In this paper, the technique is used to enhance HFECG and suppress the noise such as motion artifacts and external electromagnetic interference.

More than 100 beats of averaged HFECG segments were obtained from each measurement. The HFECG segments were divided into 70% training datasets and 30% test datasets for machine learning–based discrimination block. During machine learning, 70 training data and 30 test data were randomly obtained from this data set.

**Figure 5:**
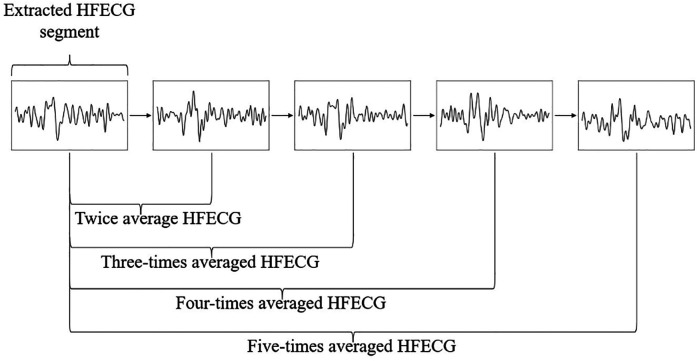
Synchronized averaging with consecutive HFECG segments.

Synchronized averaging is performed with successive 2–5 beats of HFECGs, considering the availability in practical use. Among the current methods used to open locks, opening a lock with a key takes only a few seconds. Since the minimum heart rate for a healthy person is approximately 60 beats per minute, the measurement of 5 beats is completed within approximately 5 s, which is an acceptable waiting time for the user. In our proposal, synchronized averaging was performed with 2–5 consecutive beats of the HFECG, as shown in Figure 5.

#### 2.2.4 Training data preparation

The registration process must be required for the authentication system. However, the time taken for the registration is limited due to the usability of the system. This means that an insufficient amount of training data must be used for machine learning. This may degrade the identification performance. Furthermore, the application of synchronized averaging also reduces the amount of data. In this paper, the data expansion block generates artificial HFECGs to obtain a sufficient amount of training data.

In machine classification, the artificially created data are added to the training data to improve the performance with a small amount of original training data. In image recognition, flip, rotation, and zooming in and out are used to create artificial data. In one-dimensional data, a mixture of other data or noise is often used for data augmentation (17). These techniques allow for more data to be learned, so that the system achieves satisfactory performance under a limited amount of training data.

In this paper, data augmentation was performed by mixing randomly selected HFECG segments. Data sources are non-averaged, twice averaged, and three times averaged HFECG segments. Averaging calculation is the same as test data as shown in Figure 5. In the training data augmentation tests, three patterns of data augmentations are tested as shown in Table 3. In the evaluations, three times averaged data were used for test data.

**Table 3:** Training dataset configuration for training data augmentation test.

Evaluation	Training dataset ID	Source data (number of segments)
Effect of the amount of training dataset	3avg-70TD	Three-time averaged (70)
	2,3avg-140TD	Twice averaged (70) + three times averaged (70)
	1,2,3avg-210TD	Non-averaging (70) + twice averaged (70) + three times averaged (70)
Effect of averaging for training dataset	3avg-70TD	Three-time averaged (70)
	2avg-70TD	Twice averaged (70)
	1avg-70TD	Non-averaging (70)
Effect of the combinations of averaged data for training dataset	1,2avg-140TD	Non-averaging (70) + twice averaged (70)
	1,3avg-140TD	Non-averaging (70) + three times averaged (70)
	2,3avg-140TD	Twice averaged (70) + three times averaged (70)

### 2.3 Identification performed by machine learning technique

In this paper, we focused on machine learning as a classifier. We compared a three-layered neural network and a support vector machine. These two methods are widely used for classification and show sufficient performance. In this paper, we used these machine learning methods to compare with previous studies (12–14). The system parameters are shown in Tables 4 and 5. Hyperparameters were determined by preliminary analysis. Neural network is hereinafter referred to as NN. Support vector machine is referred to as SVM. An HFECG segment, that is, 200 samples of amplitudes, is applied to the classifier as an input. One of the persons is selected from the registered subjects as an output of the classifier.

**Table 4:** NN parameters.

Hyperparameters	
Input layer size	200
Hidden layer size	100
Activation function	ReLU
Output layer size	10,7
Activation function	sigmoid
Batch size	16
Epoch	200
Optimization method	Adam(lr = 0.001)

**Table 5:** SVM parameters.

Hyperparameters	
C	1
Kernel	Linear

### 2.4 Evaluation

Identification performance was evaluated using accuracy. The confusion matrix as shown in Table 6 is widely used to evaluate classification performance. Accuracy is calculated by the equation below.

$$\mathrm{Accuracy}=\frac{\mathrm{TP}+\mathrm{TN}}{\mathrm{TP}+\mathrm{TN}+\mathrm{FP}+\mathrm{FN}}$$

**Table 6:** Confusion matrix.

Confusion matrix		Predicted	
		Positive	Negative
True	Positive	True positive (TP)	False negative (FN)
	Negative	False positive (FP)	True negative (TN)

For comparison with the previous study, the same evaluation methods were used to evaluate. In this paper, performance was tested in the registered persons. Therefore, FAR and FRR were not calculated. In each condition, 10 cycles of identification processes with randomly chosen HFECG segments were performed, and the average value of accuracy was used for comparison. This number of identification cycles was determined by considering the total number of HFECG segments extracted from the measured ECGs and the number of HFECG segments used for training and identification.

## 3 Result

### 3.1 Performance evaluation by synchronized averaging

The usefulness of the synchronized averaging for noise reduction is evaluated. The relationship between the number of the data for averaging and accuracy was calculated. The numbers are changed from 1, which is non-averaging, to 5. Averaging is applied to both training data and test data.

**Figure 6:**
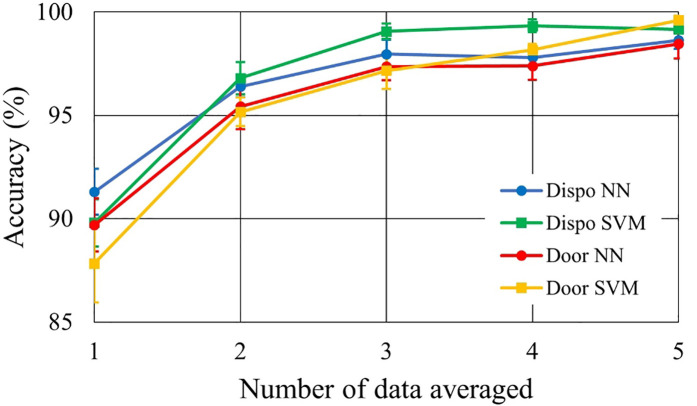
Performance improvement by synchronized averaging.

Figure 6 shows the relationship between the number of data used for synchronized averaging and accuracy for 10 subjects. This is the change in accuracy when the average number of averaging was varied from 1 to 5: “1” on the horizontal axis means no averaging. The averaged accuracies are shown in Table 7 for comparative discussion. In Figure 6 and Table 7, two types of electrodes and two types of discriminators are compared. The averaged accuracies were calculated with the results of all the individual subjects. We checked how much bias is found between subjects by using a confusion matrix. Table 8 shows a confusion matrix sample. No remarkable performance bias was found as a check result.

**Table 7:** Accuracies in different electrodes, classification method, and number of data averaged.

Number of times	1	2	3	4	5
Dispo NN	91.3%	96.4%	98.0%	97.8%	98.6%
Dispo SVM	89.8%	96.8%	99.1%	99.3%	99.2%
Door NN	89.7%	95.4%	97.4%	97.4%	98.5%
Door SVM	87.8%	95.2%	97.2%	98.2%	99.6%

**Table 8:** Confusion matrix example.

Confusion matrix											
Door NN 5 averaged		Prediction									
		0	1	2	3	4	5	6	7	8	9
True	0	28	0	0	0	0	2	0	0	0	0
	1	0	30	0	0	0	0	0	0	0	0
	2	0	0	29	0	0	0	0	0	0	1
	3	0	0	0	30	0	0	0	0	0	0
	4	0	0	0	1	29	0	0	0	0	0
	5	2	0	0	0	0	28	0	0	0	0
	6	0	0	0	0	0	0	30	0	0	0
	7	0	0	0	0	0	0	0	30	0	0
	8	0	0	0	0	0	0	0	0	30	0
	9	0	0	0	0	0	0	0	0	0	30

### 3.2 Diversity and augmentation in training dataset

In this experiment, the effect of diversity and augmentation in training datasets on identification performance was evaluated. We used various training data sets by mixing synthesized HFECG segments that were synchronized averaging up to three times. The evaluation was performed using only a doorknob ECG from 10 subjects.

Performance change in training data size is evaluated as the first step. The identification was performed by varying the number of training data from 70, 140, and 210. The contribution of synchronized averaging in training data is evaluated for the next step. The number of synchronized averaging of the training data was changed to 3, 2, and 1 in keeping the number of the training data to 70. Finally, the performance with three training datasets which are composed of HFECGs averaged using different numbers of wave segments is tested. The size of each training dataset is 140 wave segments. A test dataset for each subject was composed of thirty averaged HFECGs. Each HFECG was averaged with three consecutive HFECGs.

**Figure 7:**
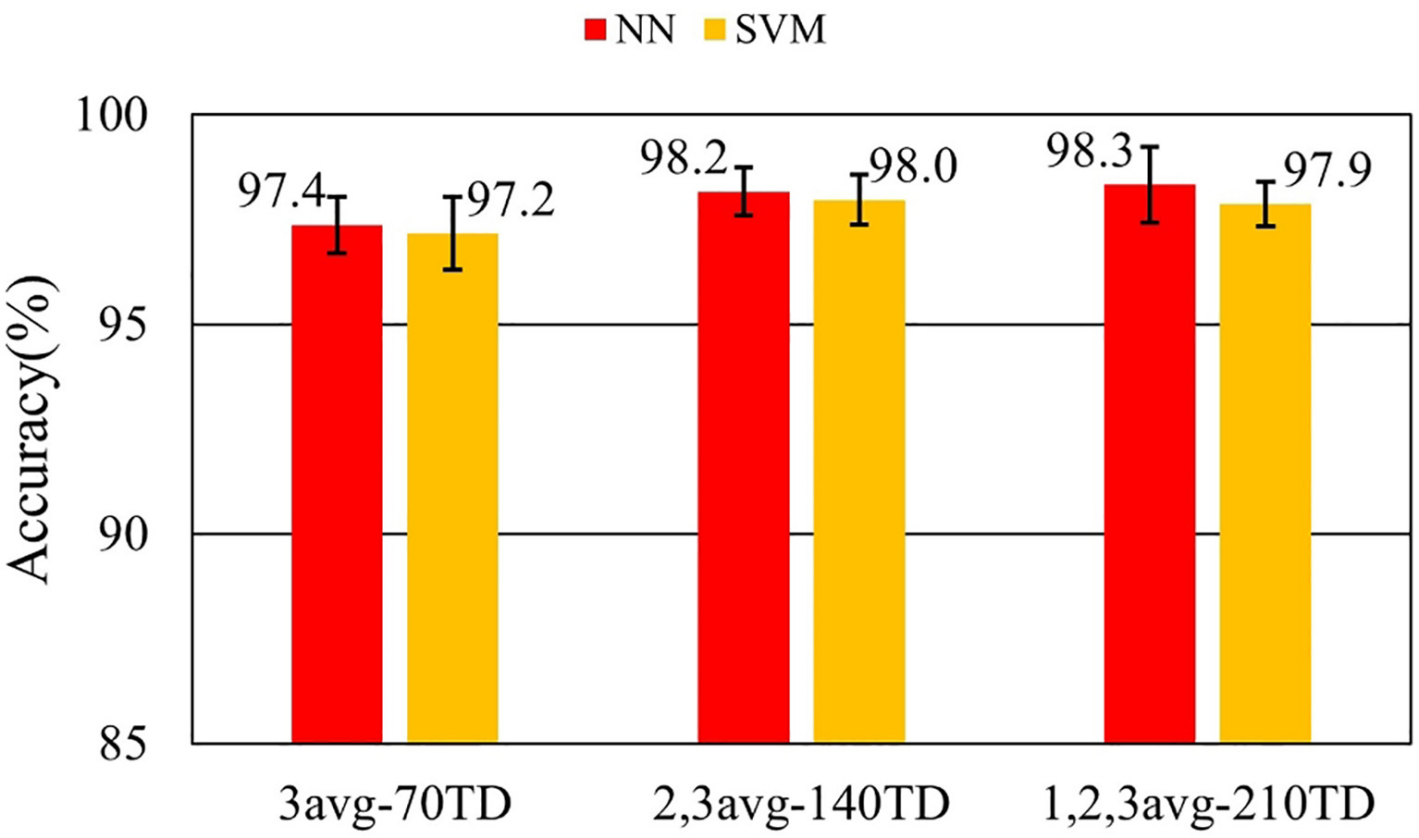
Performance in training data augmentation experiment.

**Figure 8:**
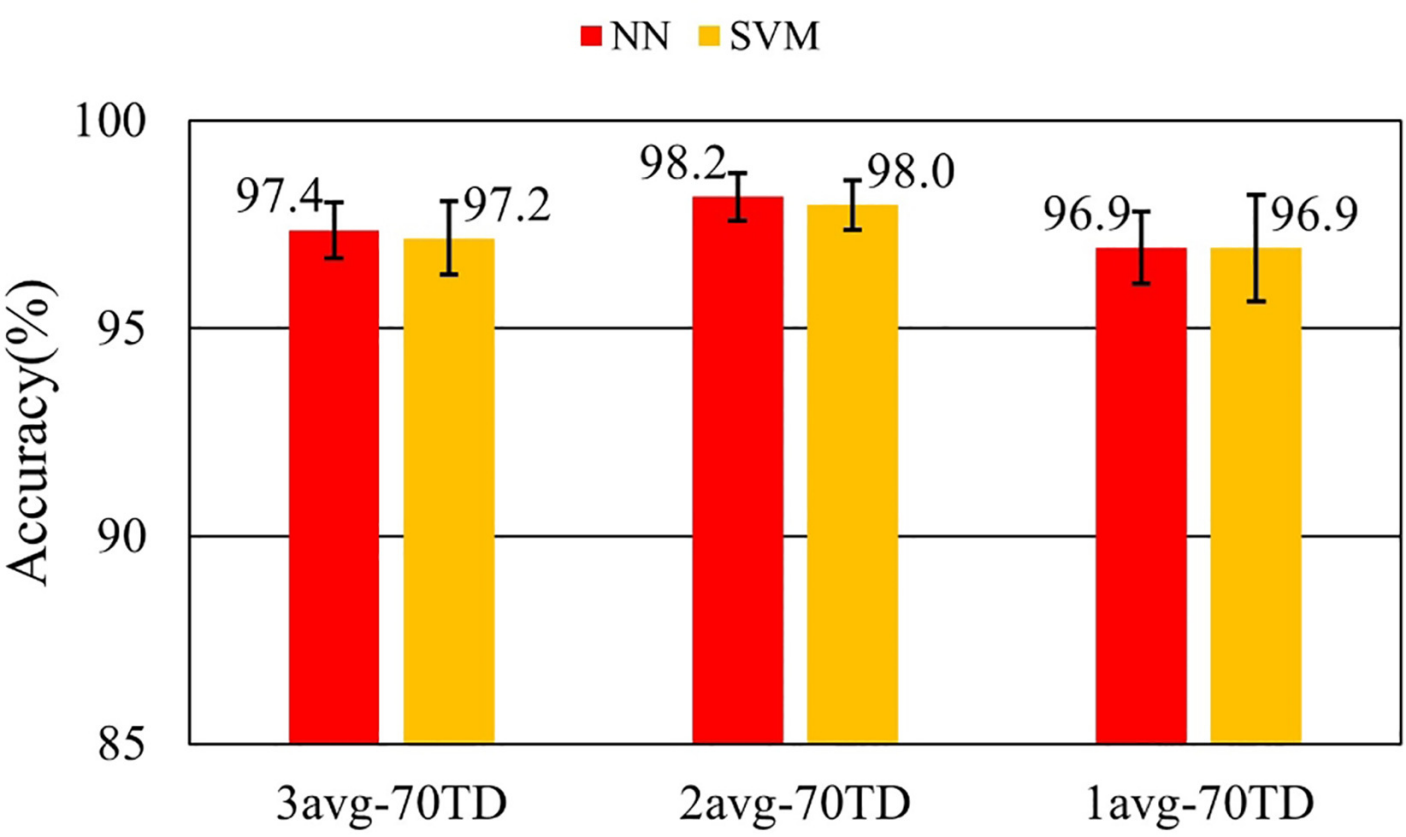
Performance with and without synchronized averaging.

**Figure 9:**
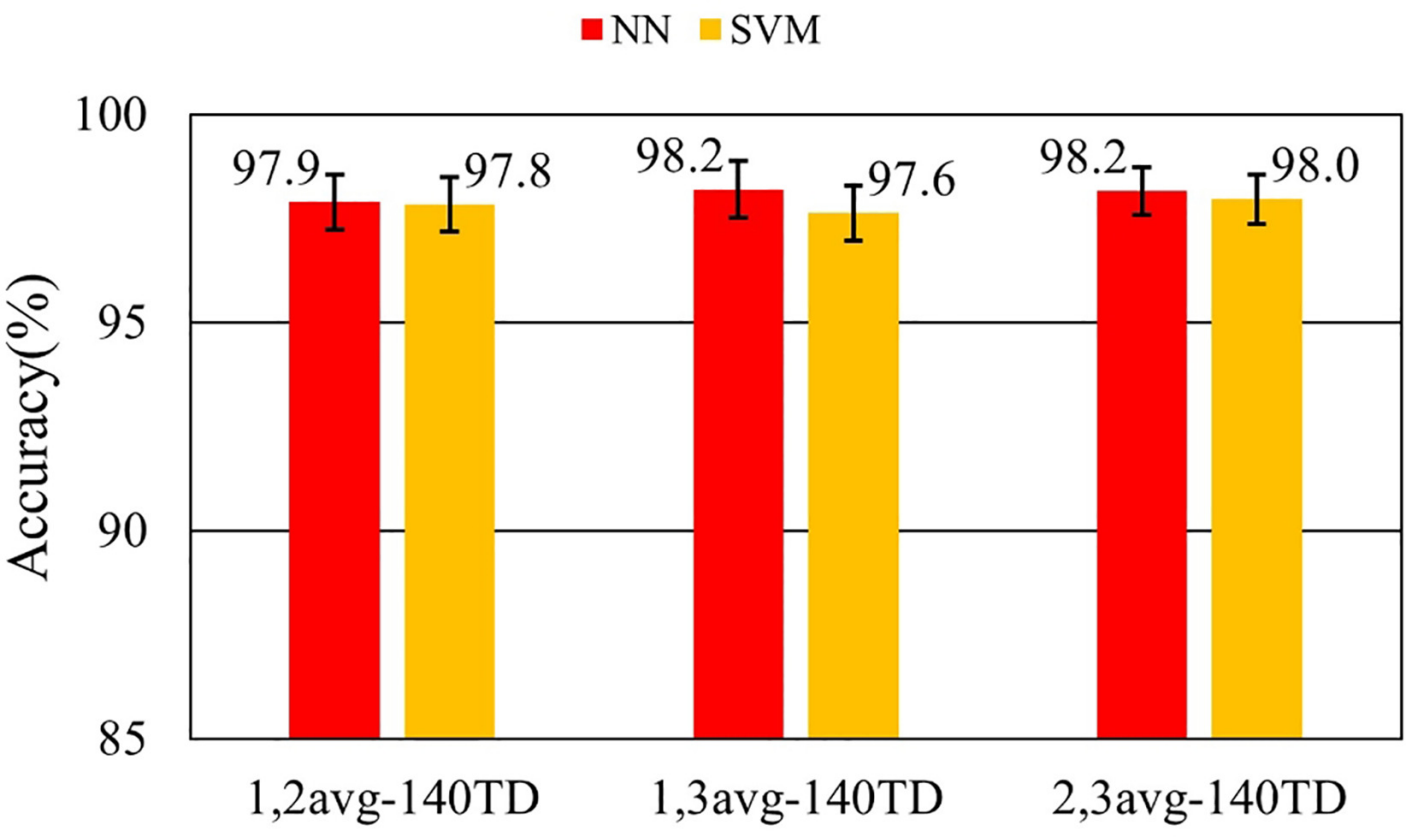
Performance in training data diversity evaluation.

Figures 7–9 and Tables 9–11 show the effect of training data extension with synchronized averaging data. The source wave segments are from original and two or three times averaged data. In Figures 7–9 and Tables 9–11, different configurations of training datasets were used as shown in Table 3. Figure 7 and Table 9 show the effect of the number of training data. Figure 8 and Table 10 show the effect of the composition of the training data. Figure 9 and Table 11 show the effect of synchronized averaging in training data.

**Table 9:** Accuracy in training data augmentation experiment.

Dataset ID	NN	NN SD	SVM	SVM SD
3avg-70TD	97.4%	0.675%	97.2%	0.878%
2,3avg-140TD	98.2%	0.572%	98.0%	0.597%
1,2,3avg-210TD	98.3%	0.903%	97.9%	0.526%

**Table 10:** Accuracy with and without synchronized averaging.

Dataset ID	NN	NN SD	SVM	SVM SD
3avg-70TD	97.4%	0.675%	97.2%	0.878%
2avg-70TD	98.2%	0.572%	98.0%	0.597%
1avg-70TD	96.9%	0.872%	96.9%	1.284%

**Table 11:** Accuracy in training data diversity evaluation.

Dataset ID	NN	NN SD	SVM	SVM SD
1,2avg-140TD	97.9%	0.668%	97.8%	0.653%
1,3avg-140TD	98.2%	0.689%	97.6%	0.656%
2,3avg-140TD	98.2%	0.572%	98.0%	0.597%

### 3.3 Reproducibility evaluation with different day’s data

**Figure 10:**
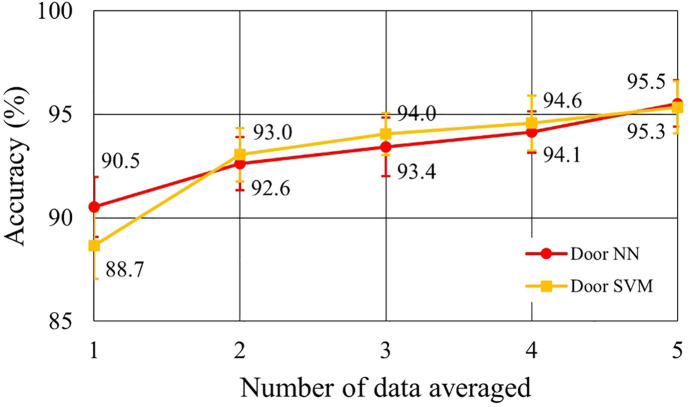
Performance improvement by synchronized averaging in reproducibility experiment.

**Figure 11:**
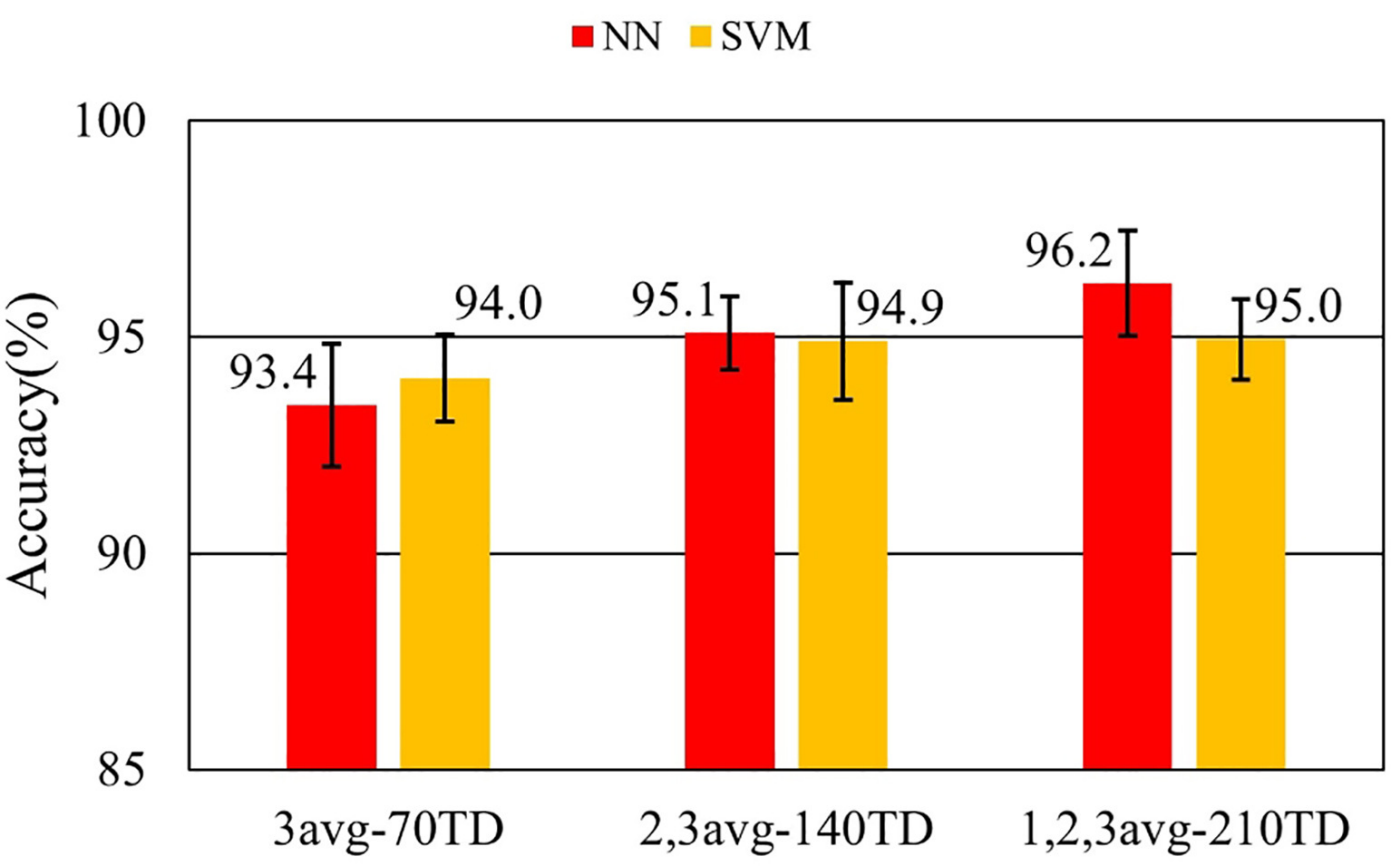
Performance with various combinations of training data in reproducibility experiment.

**Figure 12:**
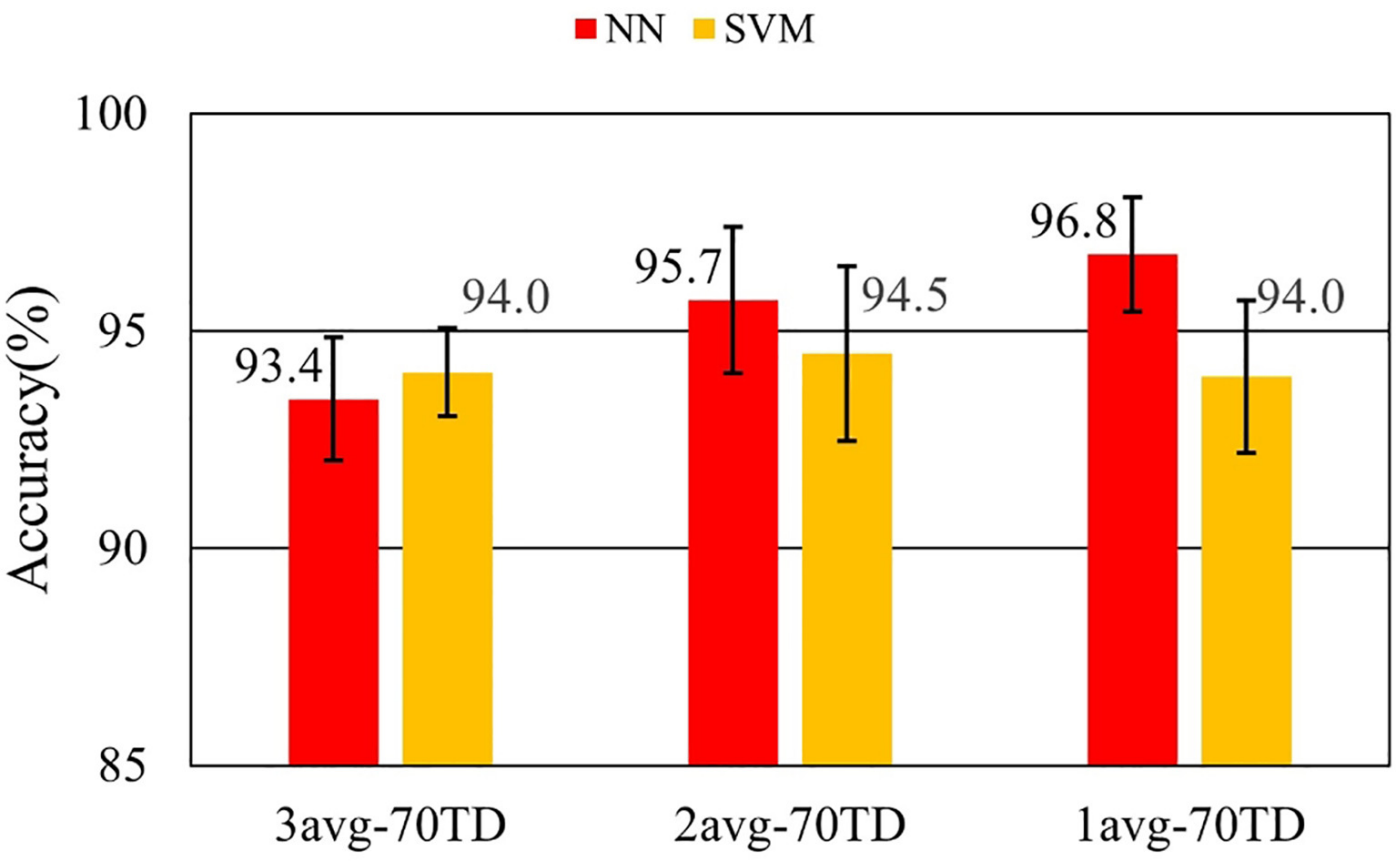
Performance with and without synchronized averaging in reproducibility experiment.

**Figure 13:**
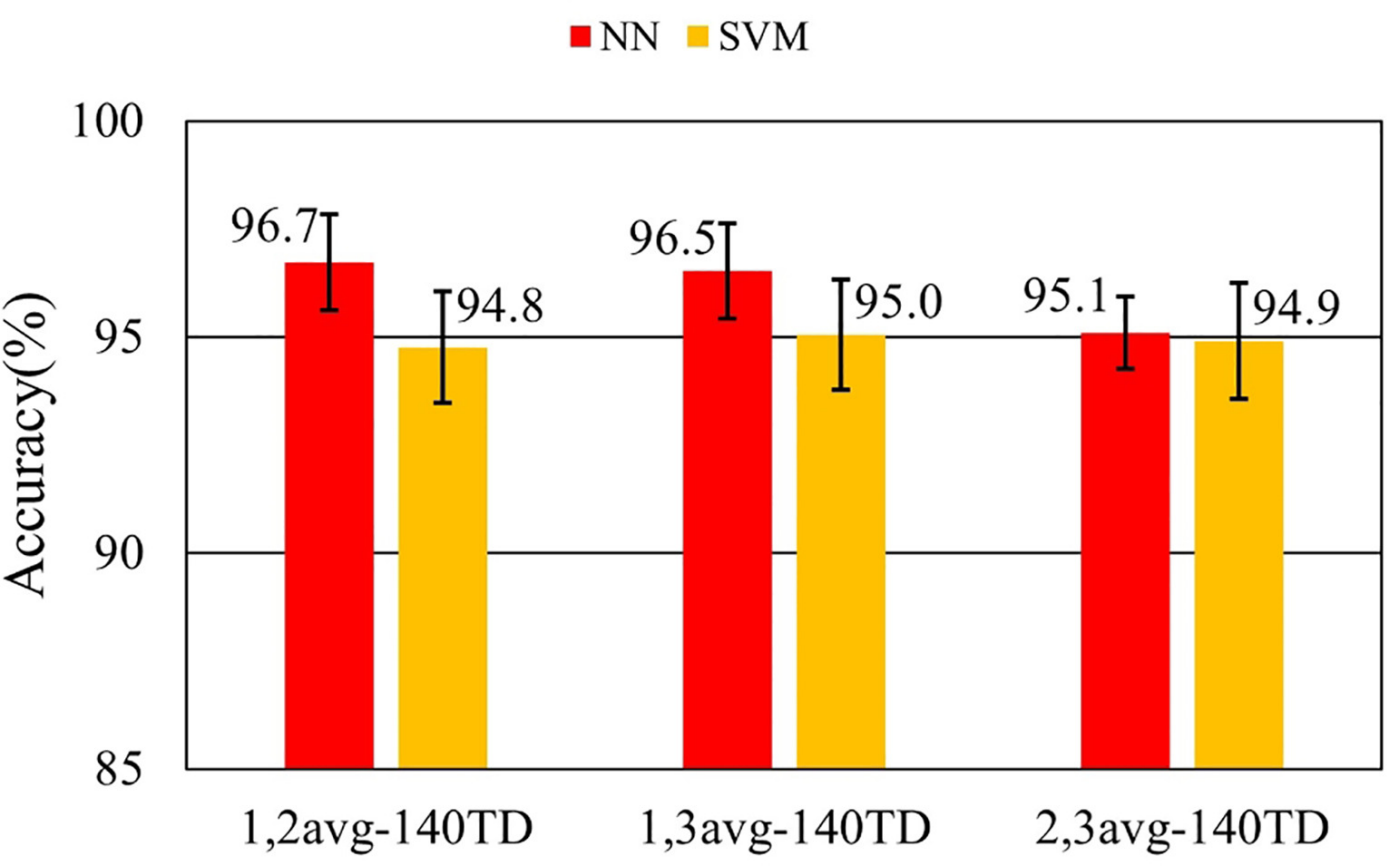
Performance in training data diversity evaluation with data from reproducibility experiment.

The classifier already trained was evaluated by applying ECG data measured on a different day as test data. Newly measured ECGs from the same 7 subjects in 10 subjects used in the previous evaluations were used for this evaluation. Figure 10 corresponds to Figure 6. In this evaluation, a doorknob ECG was only used. Figures 11–13 correspond to Figures 7–9, respectively.

## 4 Discussion

### 4.1 Performance evaluation by synchronized averaging

Figure 6 shows that synchronized averaging is effective in improving performance even in two times of averaging. This effect depends on the number of data segments for averaging. Figure 6 also shows that the accuracies in the single heartbeat waveforms were >90% in doorknob electrodes. However, in averaging five HFECG segments, the performance improved to <98% in accuracy. As shown in Table 7, the best performance for doorknob electrodes was 99.6% when using SVM with five times averaging. In this condition, SVM performance was better than that of NN. The reason may come from the classification process in SVM. When SVM determines the boundary to separate groups, it finds the optimal border where the margin between the group’s data is maximized. Synchronized averaging reduces noise in the waveform, that is, it reduces the variation of data within a group. Therefore, increased margins by averaging improves performance. It is considered in reproducible situations such as this evaluation, and SVM may show superior performance to NN.

As shown in Figure 6 and Table 7, it is found that the system performance on SVM with five HFECG segments for averaging achieves the same identification accuracy with a doorknob electrode as with a disposable electrode. Accuracy of 99.6% is a practical performance for biometric authentication systems. When this level of security is required, it takes approximately 5 s for each identification. If the level can be relaxed to 95%, the average of two beats is sufficient. Identification time can be shortened to approximately 2 s. It is practical considering the balance between the performance required and the time that can be spent on measurement.

A sample confusion matrix is shown in Table 8. The confusion matrix for Door NN when averaging five times. The distribution was similar under other conditions. This table shows that there is no bias in the differences in values due to other evaluation indices.

### 4.2 Diversity and augmentation in training dataset

Figure 7 shows that the discrimination accuracy was improved by increasing the training data. It also shows that an additional 70 training data with different synchronized averaging counts compensates for the missing features. It is considered that a well-trained classifier leads to improved accuracy. In NN, although the average accuracy at 210 training data sets is not improved by an additional 70 datasets, the standard deviation is larger. This is due to overlearning caused by adding non-averaged noisy data to training data.

Figure 8 shows the influence of noise on training data. The best accuracy was obtained by 2avg-70TD. This result is believed to be due to the best balance between diversity and low noise. In 3avg-70TD, it was considered that diversity in the training data set was lost by averaging. In 1avg-70TD, it was considered that the noise component in the training data was too large to capture the features necessary for classification.

Figure 9 shows that the combination of data with different numbers of synchronized averaging did not have a significant impact on accuracy. It suggests that the number of training data had a greater impact on accuracy than the composition of the training data.

These results demonstrate that a larger amount of training data is suitable for identification by using data augmentation in NN. Furthermore, it is suggested that an appropriate amount of noise in the training data is suitable for improving identification accuracy.

### 4.3 Reproducibility evaluation with different day’s data

Figure 10 shows the results using HFECGs measured on different days for the test data. Figure 10 shows the same trend as Figure 6, with accuracy improving as the averaging count increases. As shown in Figure 6, SVM gives better performance than NN for ECG measured on the same day. However, NN shows similar or better performance especially in 5 times averaging. This can be explained by the training and identification process in NN and SVM. In different day measurements, waveforms such as amplitude may change. This is due to differences in the thickness of the socks worn and the amount of perspiration at the time of measurement. It is mainly affected by the amplitude change, in SVM, the rigidly fixed border between groups may not give the correct answer. On the other hand, NN suppresses the effect of amplitude change because it acquires waveform characteristics during the learning process. Figure 10 also shows that noise reduction by synchronized averaging is useful for both methods.

As a comparison between Figure 11 and Figure 7, NN shows a similar trend of increasing accuracy as the amount of training data increases. This fact shows that data augmentation also improves performance even for the data on different days. Figures 12 and 13 show that higher accuracies were obtained with noisy training data. The fact demonstrates that diversity is important only for NN training. Augmentation of the training data in NN would be useful for identifying data from different days. In contrast, comparing performance between the same day and a different day, identification using SVM did not show significant improvement in all datasets. Two major reasons are considered to explain this result. The first is that the training in SVM may be optimized in all the training data conditions. The second is that the SVM classification process is not robust to waveform change.

These results show that NN gives better performance than SVM in identifying different day's ECGs. In this condition, it is found that data augmentation is important for performance improvement. In training data preparation, diversity should be ensured by mixing non-averaged and averaged data.

## 5 Conclusion

In this paper, we proposed preprocessing methods to improve performance for the purpose of a feasible personal identification system using electrocardiograms measured at doorknob electrodes. The methods employed were synchronized averaging application to HFECG wave segment and data augmentation for training data.

The results on synchronized averaging show that this technique is effective for performance improvement. In the same day's data, SVM and synchronized averaging achieved 99% accuracy for 10 subjects even with doorknob electrodes. This performance is approximately 10% better than the previous method without averaging. However, this result is not practical because measurements for authentication are usually performed on different days. In the next evaluation on reproducibility, the performance was approximately 95%. It is 4% inferior to the first result. However, it is 6% better than the performance of the previous method. The balance between usability and security level can be selected by setting the number for averaging. It also depends on the number of registrants.

The results on data augmentation show that data expansion by adding averaged data is effective not only for performance but also for saving registration time. In NN, the mixture of noisy HFECG to training data is effective for performance improvement. Considering the acquisition time, 50 s of ECG is required for 140 sets of training data which gives sufficient performance. The proposed method enabled us to realize to reduce measurement time. This performance is considered acceptable for real-world applications. The results using different day's ECGs show that data augmentation is effective only for NN and that NN gives better performance than SVM. It is sufficient performance in practical use because accuracy exceeds 95%. In our evaluations, it was shown that optimized machine learning was obtained by using 140 training data sets. It is also important for the training dataset to include raw waveforms polluted by a certain level of noise for better performance.

It is shown that our proposed system is practical not only for performance but also for usability. It can be concluded that the proposed doorknob identification system has sufficient performance as a practical biometric authentication system. In future work, other machine learning techniques such as deep learning can be used to improve performance (18). Furthermore, it is also expected that performance can be further improved by combining it with other biometric technologies such as fingerprints or photoplethysmography.

## Data Availability

The raw data supporting the conclusions of this article will be made available by the authors, without undue reservation.
