# Supplementary material for: A novel personal identification system using doorknob lead electrocardiograms for unconscious authentication in unlocking doors
Source: Front Digit Health. 2025 Jun 20;7:1585431. doi: 10.3389/fdgth.2025.1585431 (PMC12226577; doi:10.3389/fdgth.2025.1585431)
Supplement: Supplementary file 1 [file Datasheet1.pdf]

Supplementary Table 1. Performance evaluation by synchronized averaging

same day		Number of data averaged				
		1	2	3	4	5
Dispo NN	Accuracy	0.913	0.964	0.980	0.978	0.986
	Recall	0.913	0.964	0.980	0.978	0.986
	Precision	0.917	0.967	0.982	0.981	0.988
	F1	0.912	0.964	0.980	0.978	0.986
Dispo SVM	Accuracy	0.898	0.968	0.991	0.993	0.992
	Recall	0.898	0.968	0.991	0.993	0.992
	Precision	0.902	0.969	0.992	0.994	0.993
	F1	0.897	0.968	0.991	0.993	0.992
Door NN	Accuracy	0.897	0.954	0.974	0.974	0.985
	Recall	0.897	0.954	0.974	0.974	0.985
	Precision	0.901	0.956	0.975	0.976	0.985
	F1	0.895	0.954	0.974	0.974	0.985
Door SVM	Accuracy	0.878	0.952	0.972	0.982	0.996
	Recall	0.878	0.952	0.972	0.982	0.996
	Precision	0.882	0.952	0.972	0.982	0.996
	F1	0.877	0.951	0.972	0.982	0.996

Supplementary Table 2. Diversity and augmentation in training dataset

same day		Training dataset						
		3avg-70TD	2avg-70TD	1avg-70TD	1,2avg-140TD	1,3avg-140TD	2,3avg-140TD	1,2,3avg-210TD
Door NN	Accuracy	0.974	0.982	0.969	0.979	0.982	0.982	0.983
	Recall	0.974	0.982	0.969	0.979	0.982	0.982	0.983
	Precision	0.975	0.982	0.972	0.981	0.983	0.982	0.984
	F1	0.974	0.982	0.969	0.979	0.982	0.982	0.983
Door SVM	Accuracy	0.972	0.980	0.969	0.978	0.976	0.980	0.979
	Recall	0.972	0.980	0.969	0.978	0.976	0.980	0.979
	Precision	0.972	0.980	0.972	0.980	0.977	0.980	0.980
	F1	0.972	0.980	0.969	0.978	0.976	0.980	0.979

Supplementary Table 3. Performance evaluation by synchronized averaging in reproducibility experiment.

different day		Number of data averaged				
		1	2	3	4	5
Door NN	Accuracy	0.905	0.926	0.934	0.941	0.955
	Recall	0.905	0.926	0.934	0.941	0.955
	Precision	0.912	0.934	0.943	0.952	0.963
	F1	0.903	0.924	0.933	0.939	0.954
Door SVM	Accuracy	0.887	0.930	0.940	0.946	0.953
	Recall	0.887	0.930	0.940	0.946	0.953
	Precision	0.899	0.941	0.949	0.955	0.962
	F1	0.881	0.928	0.939	0.944	0.951

Supplementary Table 4. Diversity and augmentation in training dataset in reproducibility experiment.

different day		Training dataset						
		3avg-70TD	2avg-70TD	1avg-70TD	1,2avg-140TD	1,3avg-140TD	2,3avg-140TD	1,2,3avg-210TD
Door NN	Accuracy	0.934	0.957	0.968	0.967	0.965	0.951	0.962
	Recall	0.934	0.957	0.968	0.967	0.965	0.951	0.962
	Precision	0.943	0.962	0.971	0.971	0.969	0.958	0.968
	F1	0.933	0.957	0.967	0.967	0.965	0.950	0.962
Door SVM	Accuracy	0.940	0.945	0.940	0.948	0.950	0.949	0.950
	Recall	0.940	0.945	0.940	0.948	0.950	0.949	0.950
	Precision	0.949	0.953	0.949	0.955	0.957	0.957	0.957
	F1	0.939	0.943	0.937	0.946	0.949	0.948	0.948
